# Supplementary material for: The Role of the Dopamine System in Post-Stroke Mood Disorders in Newborn Rats
Source: Int J Mol Sci. 2023 Feb 6;24(4):3229. doi: 10.3390/ijms24043229 (PMC9958627; doi:10.3390/ijms24043229)
Supplement: Supplementary file 1 [file ijms-24-03229-s001.zip › ijms-2185261-supplementary.pdf]

## SUPPLEMENTARY MATERIAL

**Table S1.** Number of animals (male/female) for each experiment

| | SHAM | MCAO | MCAP+GBR |
|---|---|---|---|
| MRI | 3/4 | 7/8 | 3/3 |
| TUNEL | 3/3 | 4/4 | 3/3 |
| TH staining | 3/3 | 6/5 | N/A |
| Geotaxis | 24/27 | 30/30 | 8/9 |
| Grasp test | 25/27 | 32/29 | 8/9 |
| CRT | 14/17 | 15/16 | 6/5 |
| Tail suspension | 24/25 | 27/28 | 8/8 |
| Forced swimming test | 22/25 | 24/23 | 5/5 |
| Open field | 7/9 | 8/8 | 5/6 |
| Western blot studies | 4/4 | 4/5 | N/A |
| HPLC | 7/8 | 8/8 | N/A |
| [$^{35}$S]GTPγS binding assays | 4/6 | 5/5 | N/A |

CRT: cylinder rear test. GBR: GBR-12909. MCAO: middle cerebral artery occlusion. TH: thyroxin hydroxylase.

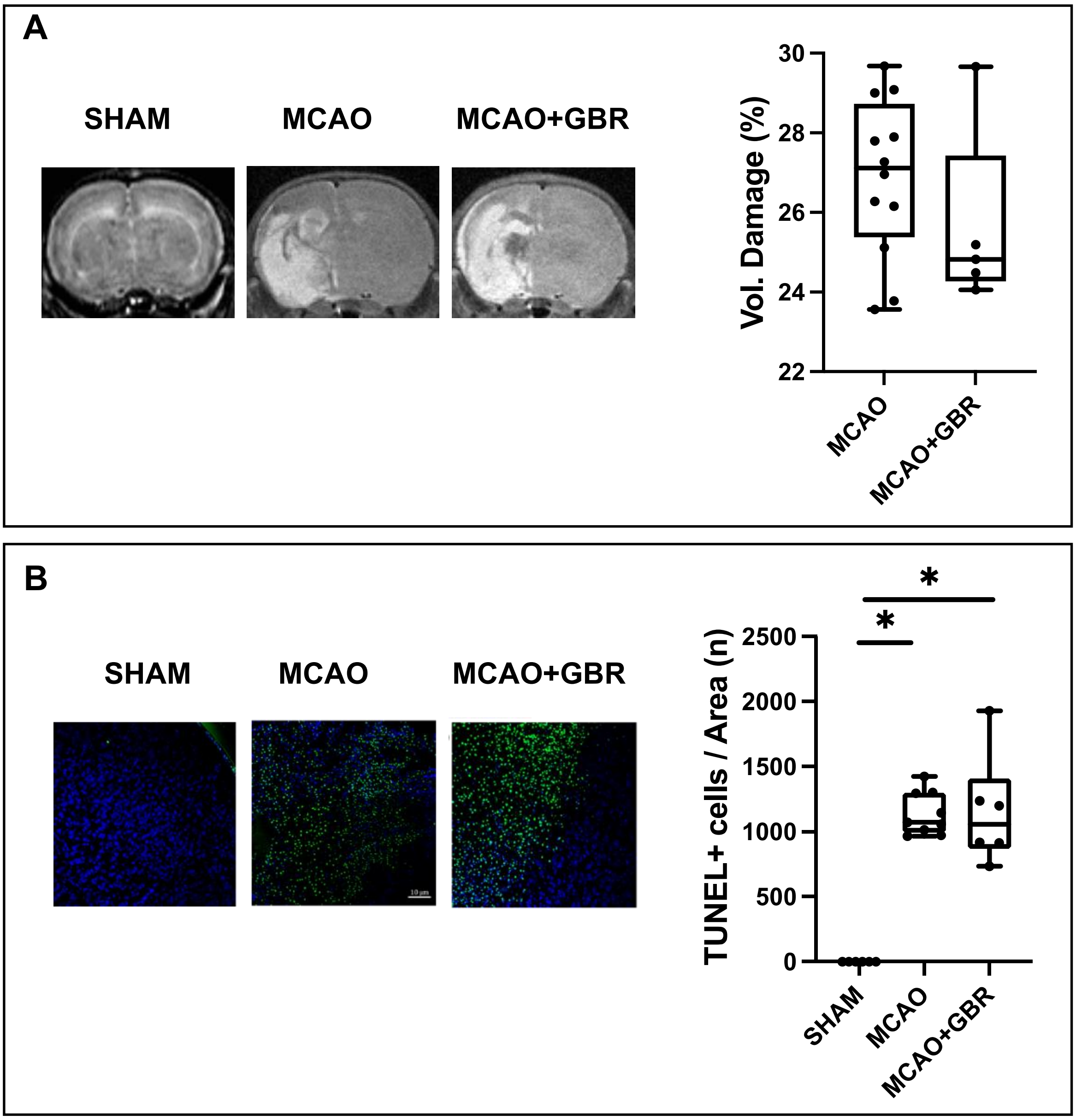


**Figure S1. Effects of the treatment with the DA reuptake inhibitor GBR-12909 on brain damage** in rats submitted to MCAO at 7 days of life (P7) and then treated with vehicle (MCAO) or GBR12909 (MCAO+GBR), and the corresponding healthy controls (SHAM). **A)** Representative T2-Weighted MRI scans, and quantification of lesion volume, measured at P14. **B)** Illustrative photomicrographs of TUNEL staining in brain samples obtained at P14, and their quantification. Original magnification: x20. Boxes represent the median and the interquartile range, while whiskers represent the minimum and maximum values in each group. CRT: cylinder rear test. A): Mann-Whitney test: U=20, p=0.32; B): (*) $p<0.05$ by Kruskal-Wallis test with Dunn's test for multiple comparisons: W=12.6, p=0.0002. Treatment with GBR-12909 did not reduce MCAO-induced brain damage.
